# Supplementary material for: Screening and Identification of a Prognostic Model of Ovarian Cancer by Combination of Transcriptomic and Proteomic Data
Source: Biomolecules. 2023 Apr 18;13(4):685. doi: 10.3390/biom13040685 (PMC10136255; doi:10.3390/biom13040685)
Supplement: Supplementary file 1 [file biomolecules-13-00685-s001.zip › biomolecules-2061181-supplementary.pdf]

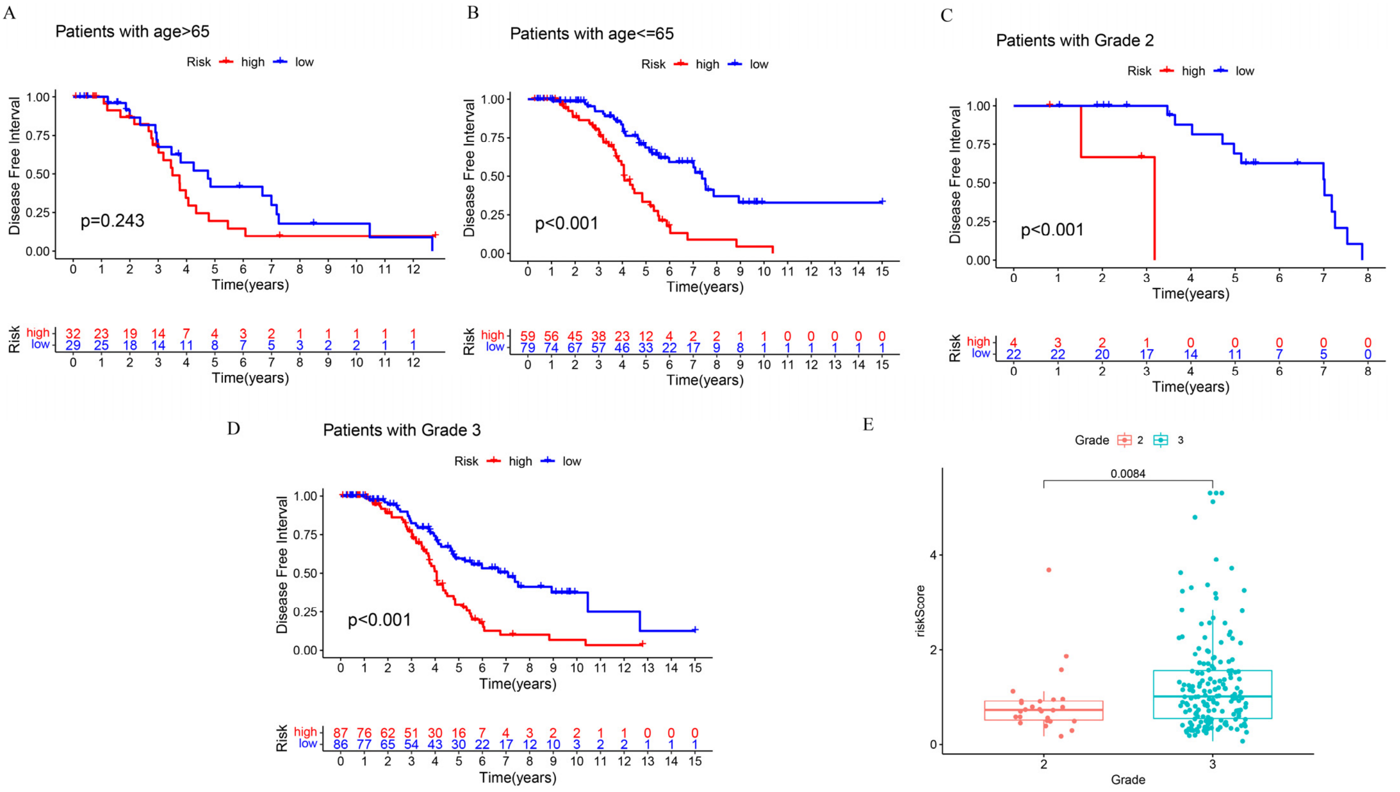

Figure S1. Correlations between prognostic-related protein risk score and clinical indicators. (A) Kaplan–Meier survival curve for disease free interval(DFI) in patients aged >65 years. (B) Kaplan–Meier survival curve for DFI in patients aged ≤65 years. (C) Kaplan–Meier survival curve for DFI in Grade 2 patients. (D) Kaplan–Meier survival curve for DFI in Grade 3 patients. (E) Scatter plot showing the correlation between risk score and grade of DFI.

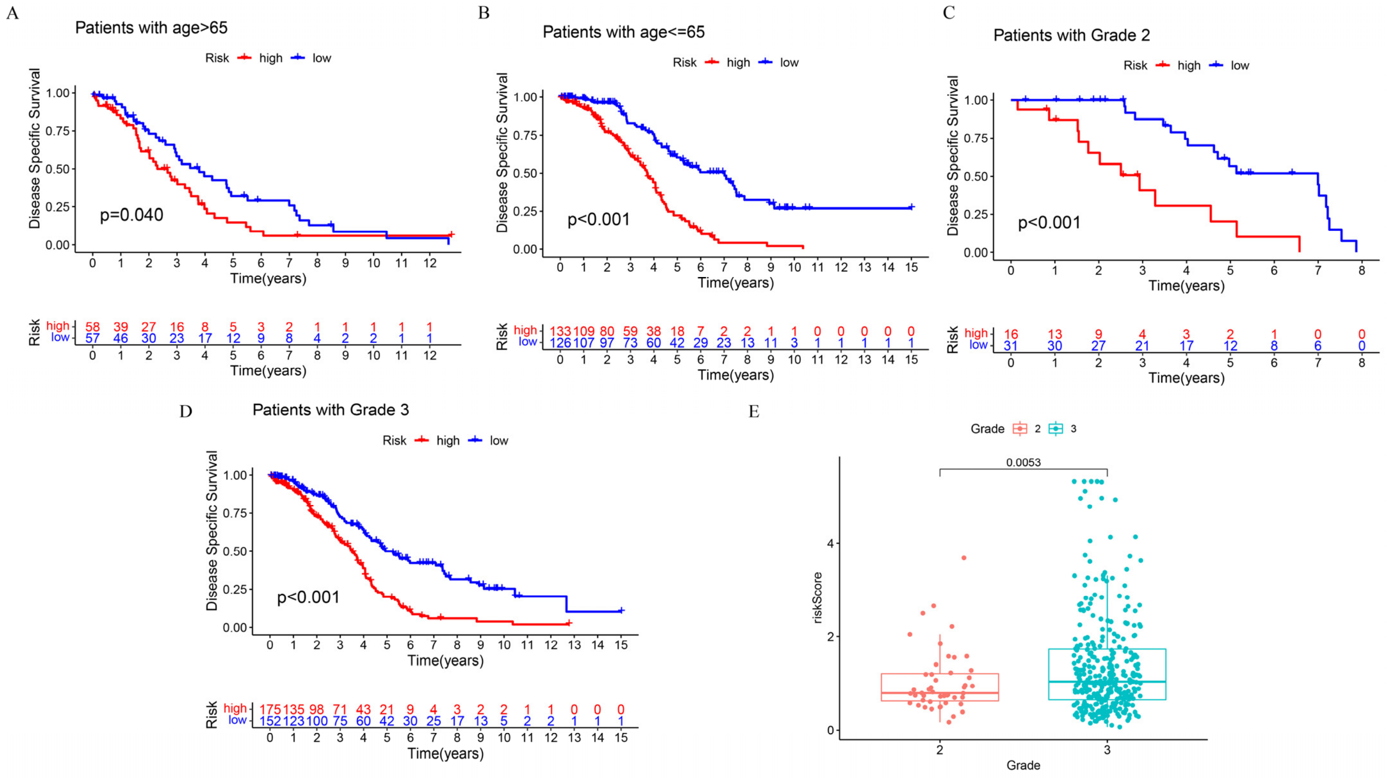

Figure S2. Correlations between prognostic-related protein risk score and clinical indicators. (A) Kaplan–Meier survival curve for disease specific survival (DSS) in patients aged >65 years. (B) Kaplan–Meier survival curve for DSS in patients aged ≤65 years. (C) Kaplan–Meier survival curve for DSS in patients with Grade 2 patients. (D) Kaplan–Meier survival curve for DSS in patients with Grade 3 patients. (E) Scatter plot showing the correlation between risk score and grade for DSS.

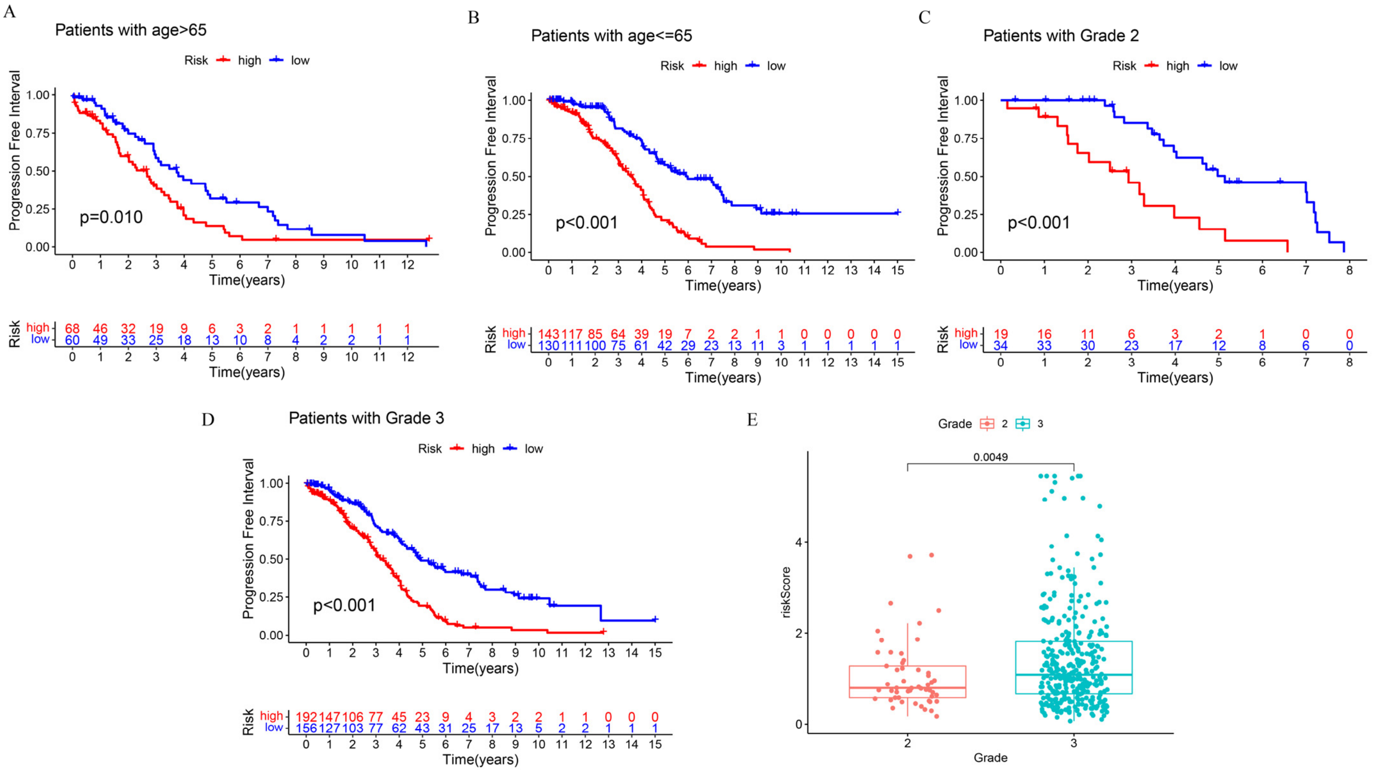

Figure S3. Correlations between prognostic-related protein risk score and clinical indicators. (A) Kaplan–Meier survival curve for progression free interval (PFI) in patients aged >65 years. (B) Kaplan–Meier survival curve for PFI in patients aged ≤65 years. (C) Kaplan–Meier survival curve for PFI in patients with Grade 2 patients. (D) Kaplan–Meier survival curve for PFI in patients with Grade 3 patients. (E) Scatter plot showing the correlation between risk score and grade to PFI.

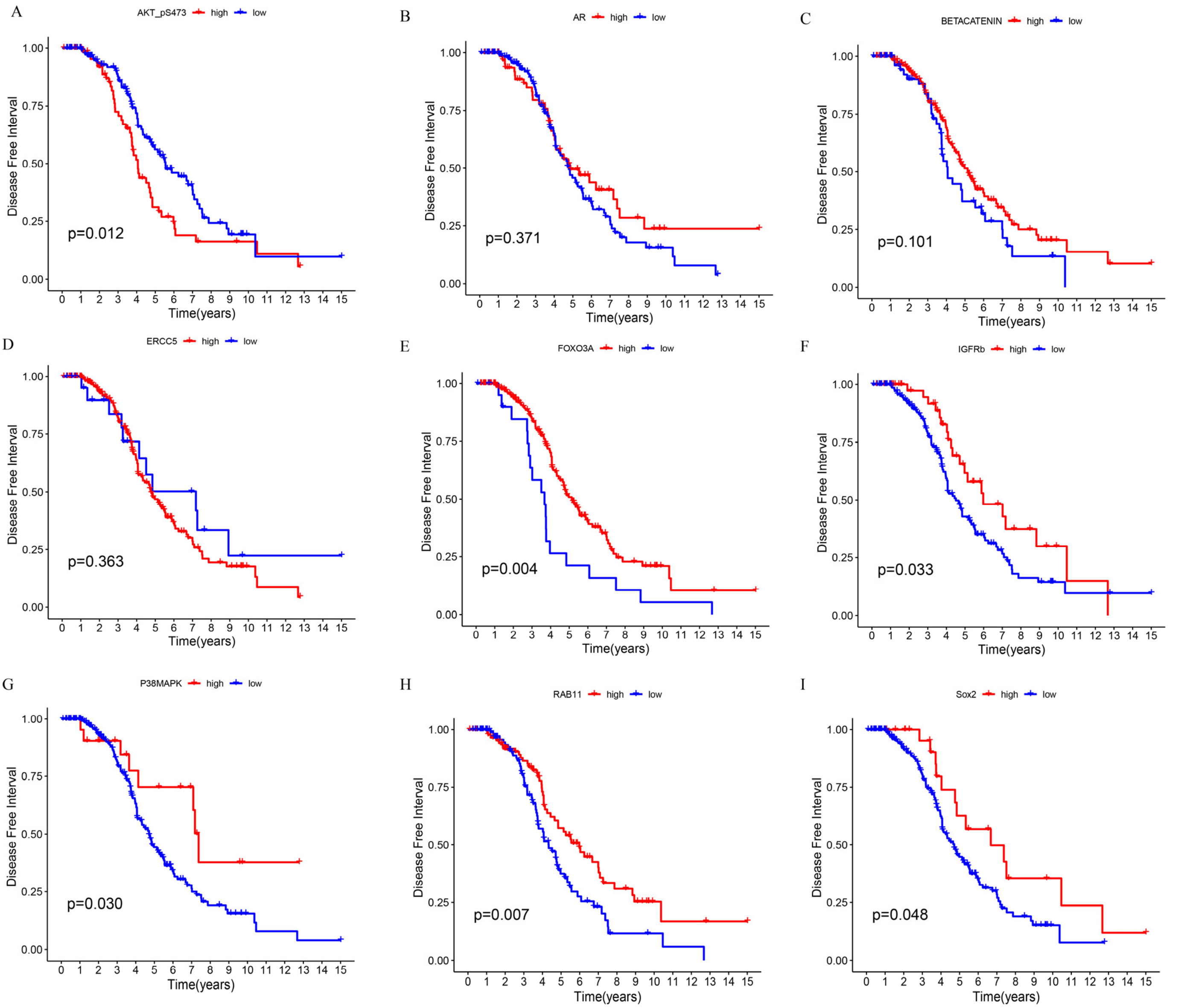

Figure S4. Kaplan–Meier survival curve of disease free interval(DFI) between high and low expression of the nine proteins' signatures.

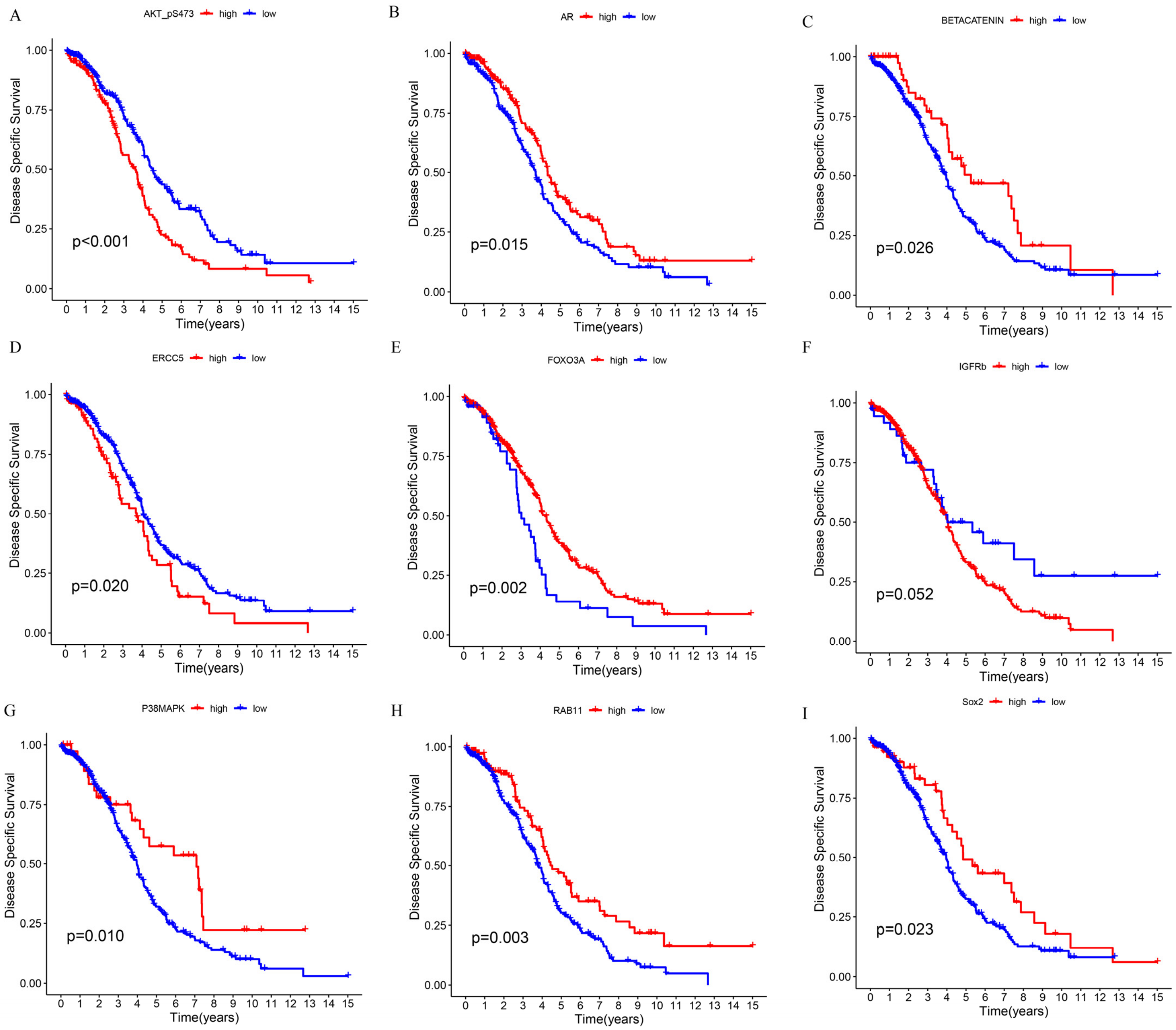

Figure S5. Kaplan–Meier survival curve of disease specific survival (DSS) between high and low expression of the nine proteins' signatures.

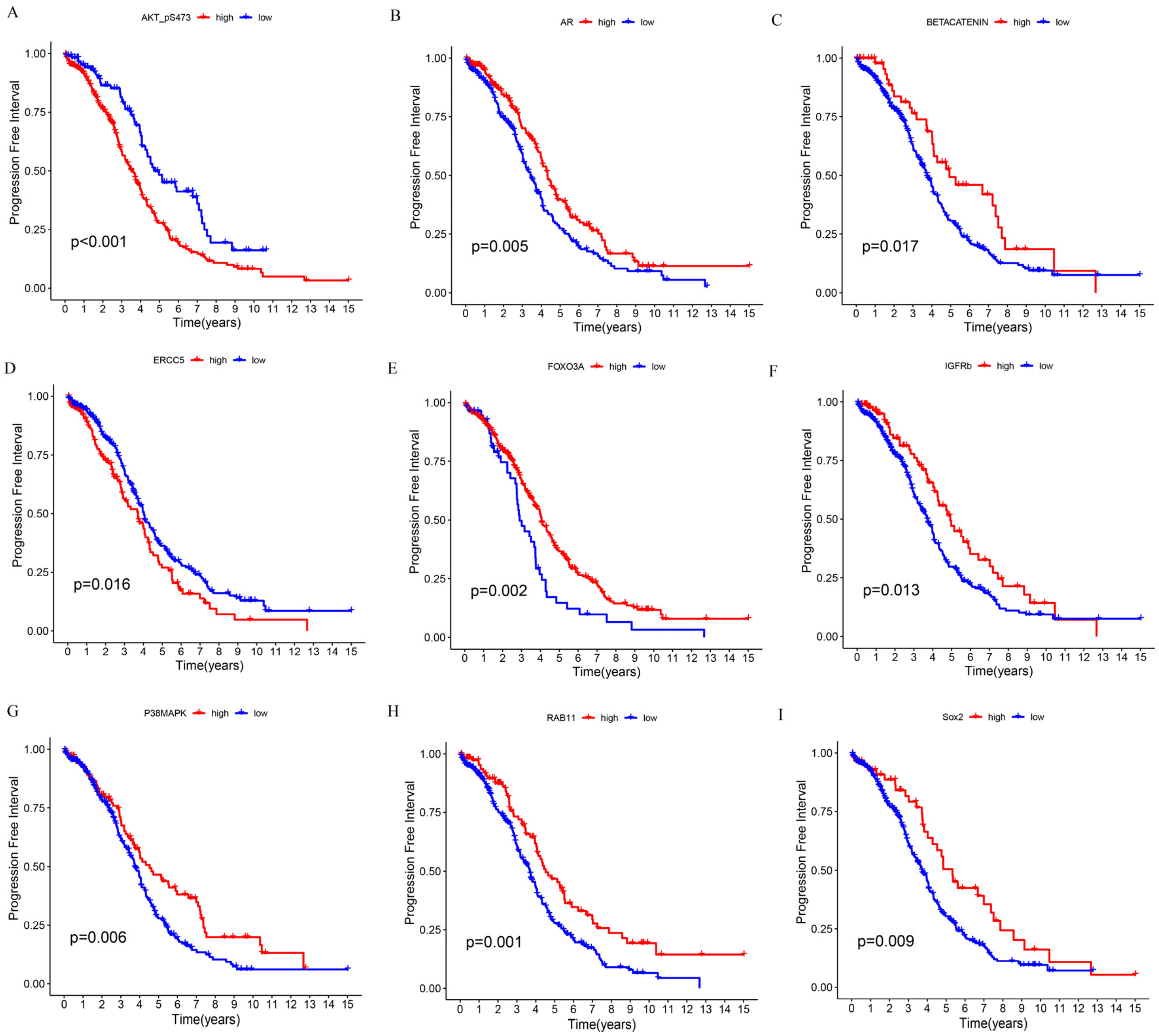

Figure S6. Kaplan–Meier survival curve of progression free interval (PFI) between high and low expression of the nine proteins' signatures.
